# Paired in vivo diagnostic accuracy and reliability of QrayCam Pro and QrayPen for occlusal and proximal caries in permanent posterior teeth

**DOI:** 10.1186/s12903-026-09756-8

**Published:** 2026-08-27

**Authors:** Elif Alkan, Ahmad Bittar, Dilek Tağtekin

**Affiliations:** 1 Department of Restorative Dentistry, Faculty of Dentistry, Marmara University, İstanbul, Türkiye; 2 Department of Restorative Dentistry, Faculty of Dentistry, İstinye University, İstanbul, Türkiye

**Keywords:** Quantitative light-induced fluorescence, QrayCam Pro, QrayPen, Occlusal caries, Proximal caries, Diagnostic accuracy, Reliability, Permanent teeth

## Abstract

**Background:**

Quantitative light-induced fluorescence (QLF) provides fluorescence-loss and red-fluorescence signals that may behave differently across devices, surfaces, and lesion thresholds. This study compared QrayCam Pro and QrayPen for occlusal and proximal caries in permanent posterior teeth.

**Methods:**

In this single-centre, paired in vivo diagnostic-accuracy and reliability study, 120 consecutively recruited adults underwent QrayPen and QrayCam Pro imaging. Multiple surfaces could be contributed by one participant. Surface-specific regions of interest in QA2 software generated maximum fluorescence loss (|ΔFmax|) and maximum red fluorescence (ΔRmax). Occlusal surfaces were classified using ICDAS and proximal surfaces using bitewing-based International Caries Classification and Management System (ICCMS)-oriented categories. Agreement was evaluated on 194 occlusal and 397 proximal paired measurements. Diagnostic analyses included 181 occlusal surfaces with complete ICDAS scores and all 397 proximal surfaces. AUC confidence intervals and paired AUC contrasts were estimated by 10,000 participant-cluster bootstrap resamples; Holm adjustment was applied across four paired contrasts within each surface-threshold family.

**Results:**

At ICDAS ≥ 1, QrayCam |ΔFmax| and QrayPen |ΔFmax| yielded AUCs of 0.909 (95% CI 0.862–0.950) and 0.872 (0.821–0.919), respectively; |ΔFmax| exceeded ΔRmax within both devices (Holm-adjusted *P* < 0.001), whereas the QrayCam-QrayPen |ΔFmax| difference did not remain significant after multiplicity correction (adjusted *P* = 0.061). At proximal ICCMS ≥ 1, AUCs ranged from 0.646 to 0.697 and no paired contrast was significant after adjustment. QrayPen |ΔFmax| exceeded QrayPen ΔRmax at ICCMS ≥ 2 (adjusted *P* = 0.022) and ≥ 3 (adjusted *P* = 0.005); at ICCMS ≥ 3, QrayCam ΔRmax exceeded QrayPen ΔRmax (adjusted *P* = 0.011). Excluding the verified QrayPen ΔRmax value of 1962 changed individual proximal AUCs by no more than 0.012.

**Conclusions:**

Metric selection influenced diagnostic performance at selected thresholds, but it was not uniformly more important than device selection. |ΔFmax| was more consistent for early occlusal detection and selected proximal thresholds, while device differences were generally small or threshold-specific. QLF should be used as an adjunct to structured visual examination and bitewing radiography rather than as a replacement.

**Trial registration:**

Not applicable; this was an observational diagnostic-accuracy study and was not prospectively registered.

**Supplementary Information:**

The online version contains supplementary material available at https://doi.org/10.1186/s12903-026-09756-8.

## Background

Contemporary caries management prioritizes early detection, biologically informed lesion assessment, and non-operative or minimally invasive care when appropriate [1–3]. Structured visual systems such as the International Caries Detection and Assessment System (ICDAS) improve clinical staging, but early or anatomically concealed lesions remain difficult to identify [4–7]. Bitewing radiography contributes information about proximal lesion depth; however, it remains an adjunct to, rather than a replacement for, clinical examination [8, 9].

Quantitative light-induced fluorescence (QLF) is a non-ionizing optical method that produces at least two conceptually distinct outputs. Fluorescence loss (ΔF) is associated mainly with changes in mineral content and optical scattering [10, 11], whereas red fluorescence (ΔR) is associated with fluorophores linked to mature biofilm and carious tissue and is affected by plaque, contamination, imaging geometry, and photobleaching [12–15]. Mechanistic studies have implicated porphyrins and advanced glycation-related fluorophores in red emission and have shown that optical signals vary with lesion chemistry [16, 17]. Accordingly, ΔF and ΔR are continuous optical signals, not direct measures of lesion activity, cavitation, sensitivity, or specificity. Lesion activity is a clinical and preferably longitudinal construct, whereas sensitivity, specificity, and area under the receiver operating characteristic curve (AUC) describe diagnostic classification relative to a stated reference standard and threshold [18, 19].

QLF has been evaluated for proximal and occlusal lesions in laboratory and clinical settings [20–24]. A Cochrane review found substantial heterogeneity across fluorescence-device studies [25], and subsequent work has addressed primary teeth, combined caries/crack detection, and inter-device field-of-view effects [26–28]. Dye-enhanced approaches have been investigated for activity-oriented assessment [29, 30], while convolutional neural networks and bibliometric analyses illustrate the expanding use of QLF images [31, 32]. Third-generation QLF has also shown clinically useful discrimination in a cross-sectional validation study [33]. A recent systematic review and meta-analysis reported stronger pooled in vivo performance for occlusal than approximal caries [34], and interventional research has used serial QLF measurements as outcomes after non-operative management [35].

Within the authors’ related research programme, separate studies have examined proximal QLF versus near-infrared imaging, full-arch versus quadrant field of view, occlusal DIAGNOdent/QrayCam comparison, and QrayPen scoring for secondary caries [36–39]. A questionnaire study evaluated examination time, interincisal distance, and tolerability [40]; it informs clinical implementation rather than diagnostic accuracy. These studies address distinct questions and are not independent validation of the present dataset.

Recent clinical work has also evaluated a new light-induced fluorescence device for occlusal caries [41]. Camera-based comparators require technological distinction: SOPROLIFE combines magnified image interpretation with fluorescence-related colour changes [42], whereas DIAGNOcam uses near-infrared light transillumination rather than fluorescence [43]. Their wavelengths, image-formation mechanisms, output scales, and thresholds are therefore not directly interchangeable with QA2-derived ΔFmax and ΔRmax.

The primary objective was to compare the diagnostic discrimination and inter-device agreement of QrayCam Pro and QrayPen for occlusal and proximal caries across defined lesion thresholds. The null hypotheses were that, at each surface type and threshold, (1) paired AUCs would not differ between the two devices for the same metric and (2) |ΔFmax| and ΔRmax would not differ within either device. The alternative hypothesis was that |ΔFmax| would show tighter inter-device agreement and more consistent discrimination than ΔRmax, while device-level differences would be smaller and threshold-dependent.

## Methods

### Study design, setting, and reporting

This was a single-centre, paired in vivo diagnostic-accuracy and reliability study conducted at the Restorative Dentistry Clinic of Marmara University Faculty of Dentistry, İstanbul, Türkiye, from July 2025 to May 2026. The report was revised in accordance with STARD 2015 and the caries-specific STARCARDDS framework [44, 45]. The protocol was not registered in a public study registry.

### Ethics approval and consent to participate

Ethical approval was obtained from the Marmara University Faculty of Dentistry Clinical and Related Studies Ethics Committee (decision date and number: 26 June 2025, 2025-07-02/2025-47; protocol no. 2025-47). All participants provided written informed consent before inclusion. The study was conducted in accordance with the Declaration of Helsinki.

### Participants, recruitment, and eligibility

Adults presenting to the clinic were screened consecutively. Eligible participants were aged 18 years or older, medically fit for routine dental examination, and had permanent first and second premolars and first molars in one arch with sound or caries-suspected occlusal and proximal surfaces. Teeth with restorations, fissure sealants, or crowns were not eligible. Exclusion criteria were extensive restorations, crowns, bridges, or orthodontic appliances involving assessed teeth; severe periodontal disease; systemic disease or medical risk precluding participation; pregnancy; inadequate oral hygiene after attempted prophylaxis; or image quality insufficient for analysis.

Of 142 adults assessed for eligibility, 22 were excluded before enrolment: 5 declined participation, 9 lacked suitable posterior teeth, 7 had plaque accumulation that could not be adequately removed during the visit, and 1 was excluded for another prespecified eligibility reason. The final sample comprised 120 adults (53 male and 67 female participants; mean age, 23.6 years). Participants could contribute multiple teeth and surfaces.

### Sample-size rationale

The ethics-approved protocol specified a minimum of 60 participants. Consecutive recruitment continued throughout the planned recruitment period to increase the number of paired evaluable surfaces, yielding 120 enrolled participants. No retrospective post hoc power calculation was performed.

### Index tests and image acquisition

The paired index tests were QrayPen and QrayCam Pro (AIOBIO Co., Ltd., Seoul, Republic of Korea). QrayPen was used for localized tooth- or surface-level imaging, whereas QrayCam Pro was used for quadrant-level imaging. Before acquisition, plaque and debris were removed, surfaces were dried with compressed air, and soft tissues were retracted as needed. Images were acquired under 405-nm blue-light illumination in accordance with the manufacturer’s operating procedures. Images that did not permit a clearly interpretable surface region were repeated during the same session.

Images were analysed using QA2 software (Inspektor Research Systems BV, Amsterdam, the Netherlands). A region of interest (ROI) was defined as the surface-specific image area selected for quantitative analysis. ROI boundaries were checked and manually corrected when required to encompass the lesion-related signal while excluding adjacent teeth, restorative materials, saliva reflections, and non-target soft tissues. Maximum fluorescence loss (ΔFmax) and maximum red fluorescence (ΔRmax) were recorded for each ROI. Signed ΔFmax values are negative when fluorescence is lost; diagnostic analyses used the absolute fluorescence-loss magnitude (|ΔFmax|) so that larger values consistently represented a stronger signal.

### Reference standards and diagnostic thresholds

Occlusal surfaces were classified clinically according to ICDAS after cleaning and air drying. Three binary thresholds were evaluated: any clinically detectable lesion (ICDAS ≥ 1), advanced enamel or dentin-related change (ICDAS ≥ 3), and dentin/cavitated involvement (ICDAS ≥ 4). Proximal surfaces were classified on bitewing radiographs using ICCMS-oriented depth categories. The collapsed categories were 0 = R0, 1 = R1-R2, 2 = R3, and 3 = R4-R6; thresholds of ICCMS ≥ 1, ≥2, and ≥ 3 represented any radiographic lesion, dentin-level involvement, and middle-or-deeper dentin involvement, respectively.

### Examiners, standardization, and masking

Three restorative-dentistry researchers participated in the assessments. EA performed the clinical ICDAS examinations. AB assessed bitewing radiographs/ICCMS categories and performed the primary Qray/QA2 measurements. DT participated as the second QLF examiner for the reliability assessment. All three had approximately 3 years of QLF research experience and prior peer-reviewed QLF work. No separate manufacturer-led calibration or certification course was undertaken. Given this prior experience, operational standardization was based on the study acquisition protocol, ICDAS/ICCMS definitions, and predefined ROI rules before formal scoring.

ICDAS, bitewing/ICCMS, and QLF/QA2 assessments were performed in separate sessions and in independently randomized image orders. The result from the other assessment was not available at the time of scoring. Because AB assessed both the radiographs and QLF images in different sessions, this procedure constituted temporal masking rather than fully independent-reader blinding.

### Reliability assessment

A simple random sample of approximately 30% of all analysed surfaces (177 of 591 pooled occlusal and proximal surfaces) was selected for repeat scoring. DT independently rescored the selected QLF/ROI measurements for inter-examiner reliability, and AB rescored the same subset in a separate session for intra-examiner repeatability. Inter-examiner reliability was assessed with a two-way random-effects, absolute-agreement, single-measure intraclass correlation coefficient (ICC); intra-examiner repeatability used a two-way mixed-effects, absolute-agreement, single-measure ICC.

### Statistical analysis

Continuous QLF values were summarized by ordinal reference category as median (minimum, maximum). Inter-device agreement was described using Bland-Altman mean differences (QrayCam minus QrayPen), 95% limits of agreement, the proportion of observations within ± 5 units, and Spearman rank correlation. Participant identifiers served as the cluster units; all tooth/surface records belonging to a resampled participant were retained together when cluster-bootstrap confidence intervals for mean differences were generated.

Diagnostic discrimination was evaluated using nonparametric receiver operating characteristic curves. AUCs and 95% confidence intervals were estimated with 10,000 participant-cluster bootstrap resamples. Four paired contrasts were evaluated within each surface-threshold family: QrayCam versus QrayPen for |ΔFmax|, QrayCam versus QrayPen for ΔRmax, |ΔFmax| versus ΔRmax within QrayPen, and |ΔFmax| versus ΔRmax within QrayCam. Two-sided bootstrap P values were adjusted by the Holm method within each family. Exploratory cutoffs were selected by the Youden index, with higher specificity used to break ties; sensitivity, specificity, accuracy, positive predictive value (PPV), and negative predictive value (NPV) were calculated at these internally derived cutoffs. They were not treated as externally validated thresholds.

All 397 proximal surfaces had complete reference-standard data. Thirteen of 194 occlusal surfaces had paired QLF measurements but no complete ordinal ICDAS score; these surfaces were retained for agreement analyses but excluded from ROC analyses without imputation, leaving 181 occlusal surfaces. The proximal QrayPen ΔRmax value of 1962 was verified against the source record and retained in the primary analysis. A sensitivity analysis repeated proximal AUC and agreement calculations after excluding that observation. Analyses were performed in Python 3.13 using pandas, scikit-learn, SciPy, and statsmodels. Statistical significance was evaluated at two-sided *P* < 0.05 after Holm adjustment.

## Results

### Participant and surface flow

**Fig. 1:**
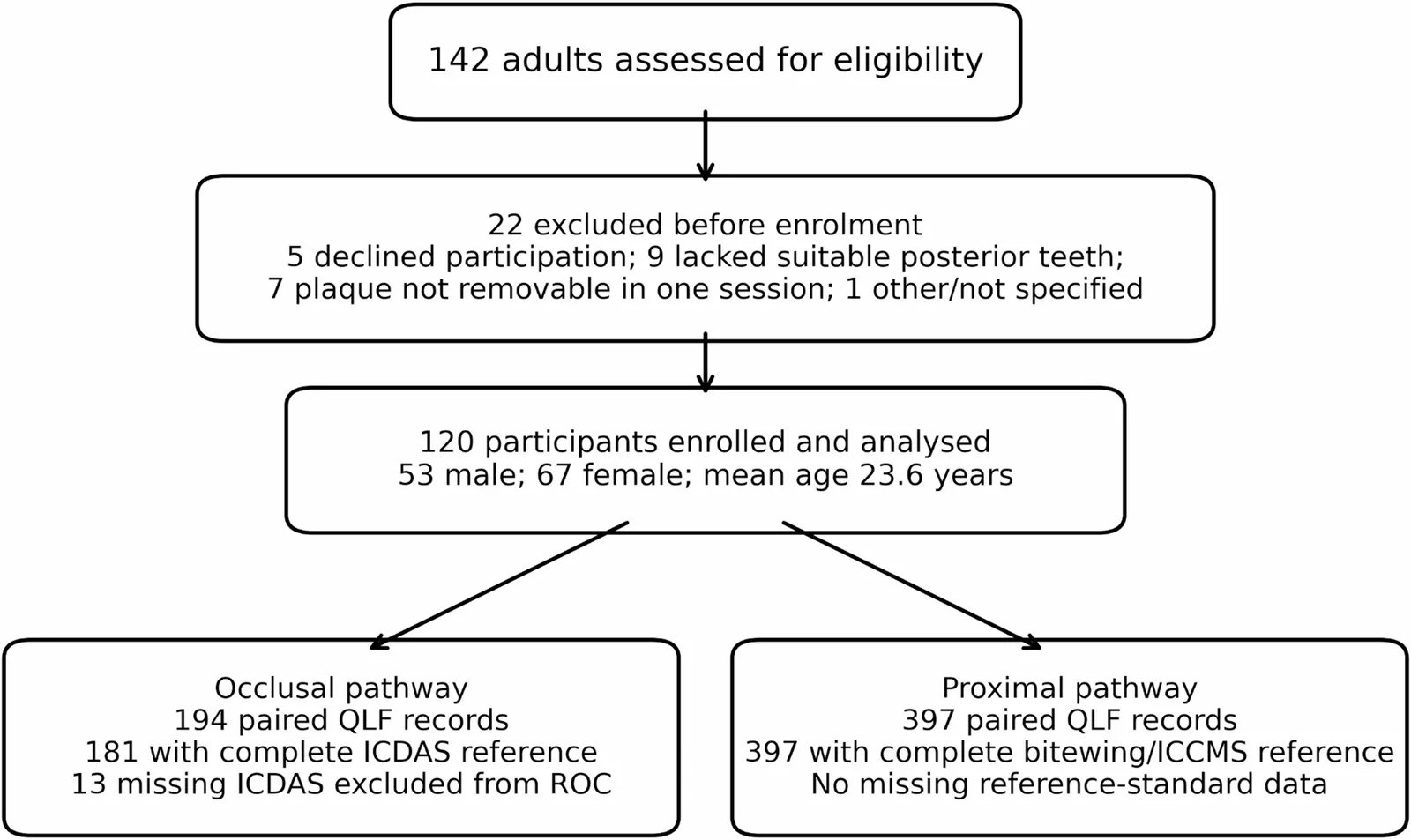
Participant-to-surface flow. The 13 occlusal surfaces without complete ordinal ICDAS scores were retained for paired device-agreement analysis but excluded from ROC analysis without imputation

The 120 enrolled adults contributed 194 paired occlusal and 397 paired proximal QLF measurements. All paired measurements were available for agreement analysis. For diagnostic accuracy, 181 occlusal surfaces had complete ICDAS reference scores (107 positive/74 negative at ICDAS ≥ 1; 47/134 at ICDAS ≥ 3; 24/157 at ICDAS ≥ 4), whereas all 397 proximal surfaces had complete ICCMS categories (131/266 at ICCMS ≥ 1; 39/358 at ICCMS ≥ 2; 21/376 at ICCMS ≥ 3). No index-test value was imputed. Participant and surface flow is shown in Fig. 1.

### Inter-device agreement and reliability

Agreement was consistently tighter for |ΔFmax| than for ΔRmax. On occlusal surfaces, the mean QrayCam-QrayPen difference was 1.15 units for |ΔFmax| and − 2.96 units for ΔRmax; the corresponding limits of agreement were − 16.30 to 18.61 and − 32.43 to 26.51. On proximal surfaces, |ΔFmax| limits were − 20.98 to 20.29, whereas ΔRmax limits widened to -197.58 to 190.15 because of the extreme QrayPen ΔRmax observation. Spearman correlations ranged from 0.793 to 0.901 (Table 1). Inter-examiner reliability was good (ICC = 0.89), and intra-examiner repeatability was excellent (ICC = 0.92). 

**Table 1:** Agreement between QrayCam Pro and QrayPen

Surface	*n*	Metric	Mean difference (95% cluster CI)	95% limits of agreement	Within ± 5	Spearman ρ
Occlusal	194	|ΔFmax|	1.15 (-0.28 to 2.63)	-16.30 to 18.61	49.5%	0.901
Occlusal	194	ΔRmax	-2.96 (-5.08 to -0.54)	-32.43 to 26.51	50.0%	0.793
Proximal	397	|ΔFmax|	-0.35 (-1.41 to 0.71)	-20.98 to 20.29	71.8%	0.831
Proximal	397	ΔRmax	-3.72 (-14.85 to 2.79)	-197.58 to 190.15	81.1%	0.784

Differences are QrayCam minus QrayPen. |ΔFmax| was analysed as fluorescence-loss magnitude. The complete Bland-Altman panels and outlier-exclusion results are shown in the supplementary material

### Occlusal diagnostic accuracy

At ICDAS ≥ 1, QrayCam |ΔFmax| had the largest AUC (0.909), followed by QrayPen |ΔFmax| (0.872), QrayCam ΔRmax (0.750), and QrayPen ΔRmax (0.703) (Table 2). |ΔFmax| exceeded ΔRmax within both QrayPen (ΔAUC = 0.169, 95% CI 0.097–0.238; Holm-adjusted *P* < 0.001) and QrayCam (ΔAUC = 0.159, 0.094–0.226; adjusted *P* < 0.001). The QrayCam-QrayPen difference for |ΔFmax| was 0.037 (0.004–0.069), but it did not remain significant after correction (adjusted *P* = 0.061) (Table 3).

**Table 2:** Paired AUC contrasts most relevant to the primary interpretation

Threshold	Paired contrast	ΔAUC	95% cluster-bootstrap CI	Unadjusted *P*	Holm-adjusted *P*
Occlusal ICDAS ≥ 1	QrayCam vs. QrayPen for |ΔFmax|	0.037	0.004 to 0.069	0.030	0.061
Occlusal ICDAS ≥ 1	QrayCam vs. QrayPen for ΔRmax	0.046	-0.003 to 0.098	0.070	0.070
Occlusal ICDAS ≥ 1	|ΔFmax| vs. ΔRmax within QrayPen	0.169	0.097 to 0.238	< 0.001	< 0.001
Occlusal ICDAS ≥ 1	|ΔFmax| vs. ΔRmax within QrayCam	0.159	0.094 to 0.226	< 0.001	< 0.001
Proximal ICCMS ≥ 1	|ΔFmax| vs. ΔRmax within QrayPen	0.050	0.008 to 0.093	0.019	0.077
Proximal ICCMS ≥ 2	|ΔFmax| vs. ΔRmax within QrayPen	0.081	0.022 to 0.147	0.006	0.022
Proximal ICCMS ≥ 3	QrayCam vs. QrayPen for ΔRmax	0.140	0.039 to 0.262	0.004	0.011
Proximal ICCMS ≥ 3	|ΔFmax| vs. ΔRmax within QrayPen	0.125	0.049 to 0.215	0.001	0.005

The full set of 24 paired contrasts is provided in Supplementary Table S4

**Table 3:** Cluster-adjusted diagnostic discrimination by surface and reference threshold

Reference threshold	Positive/negative	QrayPen |ΔFmax|	QrayPen ΔRmax	QrayCam |ΔFmax|	QrayCam ΔRmax
Occlusal ICDAS ≥ 1	107/74	0.872 (0.821–0.919)	0.703 (0.629–0.778)	0.909 (0.862–0.950)	0.750 (0.682–0.816)
Occlusal ICDAS ≥ 3	47/134	0.785 (0.699–0.861)	0.777 (0.694–0.851)	0.814 (0.732–0.887)	0.748 (0.665–0.830)
Occlusal ICDAS ≥ 4	24/157	0.785 (0.668–0.885)	0.760 (0.635–0.872)	0.795 (0.677–0.899)	0.769 (0.646–0.882)
Proximal ICCMS ≥ 1	131/266	0.697 (0.635–0.756)	0.646 (0.598–0.696)	0.692 (0.630–0.752)	0.665 (0.615–0.715)
Proximal ICCMS ≥ 2	39/358	0.847 (0.774–0.910)	0.767 (0.678–0.847)	0.851 (0.782–0.909)	0.831 (0.748–0.904)
Proximal ICCMS ≥ 3	21/376	0.892 (0.833–0.938)	0.767 (0.636–0.879)	0.908 (0.864–0.945)	0.907 (0.822–0.968)

Values are AUC (95% participant-cluster bootstrap CI). Occlusal ROC analyses included 181 surfaces with complete ICDAS scores; proximal analyses included 397 surfaces

At ICDAS ≥ 3, AUCs ranged from 0.748 to 0.814, and none of the four paired contrasts remained significant after Holm adjustment. At ICDAS ≥ 4, only 24 positive surfaces were available; AUCs ranged from 0.760 to 0.795, confidence intervals were correspondingly wider, and all paired contrasts were non-significant. These deeper-threshold estimates should therefore be interpreted cautiously.

### Proximal diagnostic accuracy

At ICCMS ≥ 1, AUCs were modest and similar: 0.697 for QrayPen |ΔFmax|, 0.692 for QrayCam |ΔFmax|, 0.665 for QrayCam ΔRmax, and 0.646 for QrayPen ΔRmax. No paired contrast was significant after Holm adjustment. At ICCMS ≥ 2, QrayPen |ΔFmax| exceeded QrayPen ΔRmax (ΔAUC = 0.081, 95% CI 0.022–0.147; adjusted *P* = 0.022), while the remaining contrasts were non-significant. At ICCMS ≥ 3, QrayCam ΔRmax exceeded QrayPen ΔRmax (ΔAUC = 0.140, 0.039–0.262; adjusted *P* = 0.011), and QrayPen |ΔFmax| exceeded QrayPen ΔRmax (ΔAUC = 0.125, 0.049–0.215; adjusted *P* = 0.005). QrayCam |ΔFmax| and QrayCam ΔRmax were essentially equivalent at this threshold (ΔAUC = 0.001; adjusted *P* = 0.956). Because only 21 surfaces were positive at ICCMS ≥ 3, these contrasts require external confirmation.

### Outlier sensitivity analysis and exploratory operating points

Excluding the verified proximal QrayPen ΔRmax value of 1962 narrowed the ΔRmax limits of agreement from − 197.58 to 190.15 to -48.60 to 50.77. Individual proximal AUCs changed by -0.002 to -0.012, indicating that the principal diagnostic-accuracy interpretation was not driven by this observation. Exploratory Youden-derived operating points and their sensitivity, specificity, accuracy, PPV, and NPV are reported in Supplementary Table S3 and should not be transferred to external populations without validation.

## Discussion

The principal contribution of this paired study is not a universal device ranking. Instead, the findings show that device, metric, surface, and lesion threshold interact. At the early occlusal threshold, |ΔFmax| produced substantially larger and statistically supported AUCs than ΔRmax within both devices, while the modest QrayCam-QrayPen difference did not remain significant after correction. At deeper occlusal thresholds, metric and device contrasts narrowed. On proximal surfaces, no adjusted contrast was significant for any radiographic lesion, but QrayPen |ΔFmax| outperformed QrayPen ΔRmax at dentin-level thresholds, and QrayCam ΔRmax exceeded QrayPen ΔRmax only at the deepest threshold. Parameter selection therefore deserves attention alongside device selection; the present data do not support the broader claim that it is uniformly more important.

The differing behaviour of ΔFmax and ΔRmax is biologically plausible. Fluorescence loss is primarily influenced by mineral-related scattering changes, whereas red fluorescence depends on fluorophores associated with biofilm and carious tissue and is additionally sensitive to plaque, contamination, acquisition geometry, and photobleaching [10–17]. A single cross-sectional ΔR value may act as a warning signal, but it does not establish lesion activity, cavitation, or the need for operative treatment. Diagnostic performance is also not an intrinsic property of the optical signal alone; sensitivity, specificity, and AUC depend on the reference standard, threshold, disease spectrum, and study setting [18, 19].

The stronger early occlusal performance of |ΔFmax| is consistent with the accessibility of clean, dried occlusal surfaces and with previous QLF work [22– [24, 28, 33]. The proximal any-lesion results were more modest, which is consistent with the optical constraints of approximal anatomy and the broader literature [20, 21, 34]. Importantly, occlusal and proximal AUCs should not be interpreted as a direct surface-type contest: ICDAS is a clinical surface-status reference, whereas bitewing/ICCMS categories describe radiographic depth. The two reference constructs overlap but are not equivalent.

The authors’ proximal study, which compared the QrayPen fluorescence-image severity score (QS) with near-infrared imaging (NIRI), reported that both modalities discriminated dentin involvement more strongly than any radiographic change [36], a stage-dependent pattern also seen here. Nevertheless, DIAGNOcam/NIRI uses near-infrared transillumination, whereas Qray devices quantify fluorescence; direct transfer of cutoffs or numeric outputs is not justified [43]. SOPROLIFE likewise depends on magnified colour interpretation rather than QA2-derived continuous ΔFmax and ΔRmax [42]. These systems may be clinically complementary, but comparisons should be made in matched populations against the same reference standard.

QLF is increasingly used as an outcome measure rather than solely as a one-time diagnostic comparator. In that context, repeated measurements of fluorescence loss, lesion area, or red fluorescence are obtained before and after non-operative management [29, 30, 35]. Such longitudinal use requires standardized acquisition, reproducible ROI placement, predefined change criteria, and evidence that observed change exceeds measurement error and corresponds to clinically meaningful lesion behaviour. The present reliability findings support repeatable measurement under the study conditions, but they do not by themselves validate a longitudinal activity threshold.

Strengths of this study include application of both devices to the same surfaces, evaluation of two biologically distinct QLF metrics, participant-clustered inference, formal paired AUC comparisons, transparent reporting of incomplete reference-standard records, and an outlier-exclusion sensitivity analysis. Moving detailed operating points and image panels to the supplementary material also clarifies the main diagnostic message.

Several limitations require emphasis. First, only 24 occlusal surfaces were positive at ICDAS ≥ 4 and 21 proximal surfaces at ICCMS ≥ 3; high AUCs at these thresholds therefore have wide uncertainty and may be unstable. Second, bitewing radiography is clinically relevant but is not a histological reference and may miss very early proximal demineralization. Third, ICDAS and bitewing/ICCMS represent different constructs, so occlusal-proximal differences cannot be attributed solely to surface anatomy. Fourth, the study was conducted at one centre in a predominantly young adult sample. Fifth, 13 occlusal surfaces lacked complete ICDAS data and were excluded from ROC analysis. Sixth, the Youden cutoffs were selected and evaluated in the same dataset and are exploratory. Seventh, radiographic and QLF scoring by the same examiner was temporally masked but not fully independent-reader blinded. Finally, the verified ΔRmax outlier markedly widened agreement limits, although its exclusion had little effect on AUCs.

## Conclusions

In this paired in vivo study, |ΔFmax| was the more consistent metric for early occlusal detection and for selected proximal dentin-level thresholds. Device differences were generally small, did not consistently survive multiplicity correction, and depended on the metric and threshold; QrayCam ΔRmax showed a specific advantage over QrayPen ΔRmax only at the deepest proximal threshold. Metric selection should therefore be considered alongside device selection rather than treated as universally more important. QLF remains an adjunctive, non-ionizing component of a diagnostic workflow led by structured visual examination and bitewing radiography.

## Supplementary Information


Supplementary Material 1.


## Data Availability

The de-identified surface-level dataset and statistical output are available from the corresponding author on reasonable request, subject to ethics approval, participant privacy, and institutional data-sharing requirements.

## Abbreviations

AUC: Area under the receiver operating characteristic curve
ICC: Inntraclass correlation coefficient
ICCMS: International Caries Classification and Management System
ICDAS: International Caries Detection and Assessment System
NIRI: Near-infrared imaging
NPV: Negative predictive value
PPV: Positive predictive value
QA2: Quantitative light-induced fluorescence image-analysis software
QLF: Quantitative light-induced fluorescence
QS: QrayPen fluorescence-image severity score
ROC: Receiver operating characteristic
ROI: Region of interest
STARD: Standards for Reporting Diagnostic Accuracy Studies
STARCARDDS: Standard Reporting of Caries Detection and Diagnostic Studies
ΔF: Fluorescence loss
ΔR: Red fluorescence
