# Supplementary material for: Paired in vivo diagnostic accuracy and reliability of QrayCam Pro and QrayPen for occlusal and proximal caries in permanent posterior teeth
Source: BMC Oral Health. 2026 Aug 27;26:1638. doi: 10.1186/s12903-026-09756-8 (PMC13527916; doi:10.1186/s12903-026-09756-8)
Supplement: Supplementary file 1 [file 12903_2026_9756_MOESM1_ESM.docx]

**Supplementary material**

Paired in vivo diagnostic accuracy and reliability of QrayCam Pro and QrayPen for occlusal and proximal caries in permanent posterior teeth

Submission ID: 85a41304-16a0-4425-bdfb-a60f2d5f8415

**Supplementary Table S1. Occlusal QLF values by ordinal ICDAS score**

| **ICDAS** | **n** | **QrayPen ΔFmax** | **QrayPen ΔRmax** | **QrayCam ΔFmax** | **QrayCam ΔRmax** |
| --- | --- | --- | --- | --- | --- |
| 0 | 74 | -9.0 (-56.0, -1.0) | 4.5 (0.0, 87.0) | -7.5 (-49.0, 0.0) | 0.0 (0.0, 57.0) |
| 1 | 31 | -29.0 (-58.0, -1.0) | 3.0 (0.0, 76.0) | -27.0 (-53.0, 0.0) | 0.0 (0.0, 52.0) |
| 2 | 29 | -38.0 (-58.0, -5.0) | 30.0 (0.0, 91.0) | -38.0 (-65.0, -16.0) | 40.0 (0.0, 124.0) |
| 3 | 23 | -42.0 (-70.0, -2.0) | 39.0 (1.0, 136.0) | -46.0 (-74.0, 0.0) | 43.0 (0.0, 131.0) |
| 4 | 22 | -49.5 (-84.0, -1.0) | 72.0 (1.0, 113.0) | -58.5 (-75.0, 0.0) | 69.0 (0.0, 113.0) |
| 5 | 2 | -37.5 (-46.0, -29.0) | 2.0 (1.0, 3.0) | -32.5 (-52.0, -13.0) | 0.0 (0.0, 0.0) |

*Values are median (minimum, maximum). Signed ΔFmax values are shown; negative values indicate fluorescence loss. Thirteen surfaces without complete ordinal ICDAS scores are not included in this descriptive table.*

**Supplementary Table S2. Proximal QLF values by ICCMS-oriented category**

| **ICCMS category** | **n** | **QrayPen ΔFmax** | **QrayPen ΔRmax** | **QrayCam ΔFmax** | **QrayCam ΔRmax** |
| --- | --- | --- | --- | --- | --- |
| 0 | 266 | 0.0 (-54.0, 0.0) | 0.0 (0.0, 66.0) | 0.0 (-49.0, 0.0) | 0.0 (0.0, 84.0) |
| 1 | 92 | 0.0 (-65.0, 0.0) | 0.0 (0.0, 88.0) | 0.0 (-53.0, 0.0) | 0.0 (0.0, 170.0) |
| 2 | 18 | -26.0 (-89.0, 0.0) | 33.5 (0.0, 430.0) | -24.5 (-54.0, 0.0) | 34.0 (0.0, 150.0) |
| 3 | 21 | -34.0 (-96.0, -8.0) | 45.0 (0.0, 1962.0) | -33.0 (-58.0, -13.0) | 64.0 (0.0, 162.0) |

*Values are median (minimum, maximum). Categories were collapsed as 0=R0, 1=R1-R2, 2=R3, and 3=R4-R6.*

**Supplementary Table S3. Exploratory Youden operating-point metrics**

| **Threshold** | **Metric** | **Cutoff** | **Sensitivity** | **Specificity** | **Accuracy** | **PPV** | **NPV** |
| --- | --- | --- | --- | --- | --- | --- | --- |
| Occlusal ICDAS ≥1 | QrayPen \|ΔFmax\| | 22 | 0.841 | 0.865 | 0.851 | 0.900 | 0.790 |
| Occlusal ICDAS ≥1 | QrayPen ΔRmax | 9 | 0.542 | 0.905 | 0.691 | 0.892 | 0.578 |
| Occlusal ICDAS ≥1 | QrayCam \|ΔFmax\| | 22 | 0.850 | 0.851 | 0.851 | 0.892 | 0.797 |
| Occlusal ICDAS ≥1 | QrayCam ΔRmax | 30 | 0.542 | 0.959 | 0.713 | 0.951 | 0.592 |
| Occlusal ICDAS ≥3 | QrayPen \|ΔFmax\| | 42 | 0.574 | 0.881 | 0.801 | 0.628 | 0.855 |
| Occlusal ICDAS ≥3 | QrayPen ΔRmax | 32 | 0.660 | 0.813 | 0.773 | 0.554 | 0.872 |
| Occlusal ICDAS ≥3 | QrayCam \|ΔFmax\| | 39 | 0.660 | 0.843 | 0.796 | 0.596 | 0.876 |
| Occlusal ICDAS ≥3 | QrayCam ΔRmax | 35 | 0.660 | 0.799 | 0.762 | 0.534 | 0.870 |
| Occlusal ICDAS ≥4 | QrayPen \|ΔFmax\| | 27 | 0.875 | 0.624 | 0.657 | 0.263 | 0.970 |
| Occlusal ICDAS ≥4 | QrayPen ΔRmax | 32 | 0.750 | 0.758 | 0.757 | 0.321 | 0.952 |
| Occlusal ICDAS ≥4 | QrayCam \|ΔFmax\| | 41 | 0.708 | 0.783 | 0.773 | 0.333 | 0.946 |
| Occlusal ICDAS ≥4 | QrayCam ΔRmax | 36 | 0.750 | 0.758 | 0.757 | 0.321 | 0.952 |
| Proximal ICCMS ≥1 | QrayPen \|ΔFmax\| | 9 | 0.573 | 0.793 | 0.720 | 0.577 | 0.790 |
| Proximal ICCMS ≥1 | QrayPen ΔRmax | 30 | 0.336 | 0.951 | 0.748 | 0.772 | 0.744 |
| Proximal ICCMS ≥1 | QrayCam \|ΔFmax\| | 23 | 0.473 | 0.898 | 0.758 | 0.697 | 0.776 |
| Proximal ICCMS ≥1 | QrayCam ΔRmax | 29 | 0.397 | 0.917 | 0.746 | 0.703 | 0.755 |
| Proximal ICCMS ≥2 | QrayPen \|ΔFmax\| | 6 | 0.897 | 0.693 | 0.713 | 0.241 | 0.984 |
| Proximal ICCMS ≥2 | QrayPen ΔRmax | 30 | 0.615 | 0.908 | 0.879 | 0.421 | 0.956 |
| Proximal ICCMS ≥2 | QrayCam \|ΔFmax\| | 16 | 0.872 | 0.796 | 0.804 | 0.318 | 0.983 |
| Proximal ICCMS ≥2 | QrayCam ΔRmax | 30 | 0.744 | 0.883 | 0.869 | 0.408 | 0.969 |
| Proximal ICCMS ≥3 | QrayPen \|ΔFmax\| | 8 | 1.000 | 0.694 | 0.710 | 0.154 | 1.000 |
| Proximal ICCMS ≥3 | QrayPen ΔRmax | 34 | 0.619 | 0.894 | 0.879 | 0.245 | 0.977 |
| Proximal ICCMS ≥3 | QrayCam \|ΔFmax\| | 23 | 0.952 | 0.816 | 0.824 | 0.225 | 0.997 |
| Proximal ICCMS ≥3 | QrayCam ΔRmax | 30 | 0.905 | 0.862 | 0.864 | 0.268 | 0.994 |

*Cutoffs were selected and evaluated in the same dataset and are therefore internal/exploratory, not externally validated.*

**Supplementary Table S4. Full paired AUC comparisons**

| **Threshold** | **Paired contrast** | **ΔAUC** | **95% cluster-bootstrap CI** | **Unadjusted P** | **Holm-adjusted P** |
| --- | --- | --- | --- | --- | --- |
| Occlusal ICDAS ≥1 | QrayCam vs QrayPen for \|ΔFmax\| | 0.037 | 0.004 to 0.069 | 0.030 | 0.061 |
| Occlusal ICDAS ≥1 | QrayCam vs QrayPen for ΔRmax | 0.046 | -0.003 to 0.098 | 0.070 | 0.070 |
| Occlusal ICDAS ≥1 | \|ΔFmax\| vs ΔRmax within QrayPen | 0.169 | 0.097 to 0.238 | <0.001 | <0.001 |
| Occlusal ICDAS ≥1 | \|ΔFmax\| vs ΔRmax within QrayCam | 0.159 | 0.094 to 0.226 | <0.001 | <0.001 |
| Occlusal ICDAS ≥3 | QrayCam vs QrayPen for \|ΔFmax\| | 0.029 | -0.008 to 0.067 | 0.118 | 0.355 |
| Occlusal ICDAS ≥3 | QrayCam vs QrayPen for ΔRmax | -0.029 | -0.072 to 0.014 | 0.182 | 0.365 |
| Occlusal ICDAS ≥3 | \|ΔFmax\| vs ΔRmax within QrayPen | 0.008 | -0.063 to 0.082 | 0.828 | 0.828 |
| Occlusal ICDAS ≥3 | \|ΔFmax\| vs ΔRmax within QrayCam | 0.066 | 0.008 to 0.128 | 0.028 | 0.112 |
| Occlusal ICDAS ≥4 | QrayCam vs QrayPen for \|ΔFmax\| | 0.011 | -0.038 to 0.059 | 0.620 | 1.000 |
| Occlusal ICDAS ≥4 | QrayCam vs QrayPen for ΔRmax | 0.009 | -0.026 to 0.050 | 0.632 | 1.000 |
| Occlusal ICDAS ≥4 | \|ΔFmax\| vs ΔRmax within QrayPen | 0.025 | -0.077 to 0.133 | 0.675 | 1.000 |
| Occlusal ICDAS ≥4 | \|ΔFmax\| vs ΔRmax within QrayCam | 0.026 | -0.048 to 0.103 | 0.512 | 1.000 |
| Proximal ICCMS ≥1 | QrayCam vs QrayPen for \|ΔFmax\| | -0.005 | -0.034 to 0.024 | 0.769 | 0.769 |
| Proximal ICCMS ≥1 | QrayCam vs QrayPen for ΔRmax | 0.019 | -0.010 to 0.049 | 0.212 | 0.442 |
| Proximal ICCMS ≥1 | \|ΔFmax\| vs ΔRmax within QrayPen | 0.050 | 0.008 to 0.093 | 0.019 | 0.077 |
| Proximal ICCMS ≥1 | \|ΔFmax\| vs ΔRmax within QrayCam | 0.027 | -0.010 to 0.065 | 0.147 | 0.442 |
| Proximal ICCMS ≥2 | QrayCam vs QrayPen for \|ΔFmax\| | 0.003 | -0.039 to 0.047 | 0.893 | 1.000 |
| Proximal ICCMS ≥2 | QrayCam vs QrayPen for ΔRmax | 0.065 | -0.017 to 0.148 | 0.115 | 0.344 |
| Proximal ICCMS ≥2 | \|ΔFmax\| vs ΔRmax within QrayPen | 0.081 | 0.022 to 0.147 | 0.006 | 0.022 |
| Proximal ICCMS ≥2 | \|ΔFmax\| vs ΔRmax within QrayCam | 0.019 | -0.034 to 0.081 | 0.510 | 1.000 |
| Proximal ICCMS ≥3 | QrayCam vs QrayPen for \|ΔFmax\| | 0.016 | -0.020 to 0.056 | 0.393 | 0.785 |
| Proximal ICCMS ≥3 | QrayCam vs QrayPen for ΔRmax | 0.140 | 0.039 to 0.262 | 0.004 | 0.011 |
| Proximal ICCMS ≥3 | \|ΔFmax\| vs ΔRmax within QrayPen | 0.125 | 0.049 to 0.215 | 0.001 | 0.005 |
| Proximal ICCMS ≥3 | \|ΔFmax\| vs ΔRmax within QrayCam | 0.001 | -0.055 to 0.068 | 0.956 | 0.956 |

*Holm adjustment was applied to the four paired contrasts within each surface-threshold family.*

**Supplementary Table S5. Sensitivity analysis excluding the proximal QrayPen ΔRmax value of 1962**

| **Threshold** | **Metric** | **Primary AUC** | **AUC without outlier** | **Change** |
| --- | --- | --- | --- | --- |
| Proximal ICCMS ≥1 | QrayPen \|ΔFmax\| | 0.697 | 0.694 | -0.002 |
| Proximal ICCMS ≥1 | QrayPen ΔRmax | 0.646 | 0.644 | -0.003 |
| Proximal ICCMS ≥1 | QrayCam \|ΔFmax\| | 0.692 | 0.690 | -0.002 |
| Proximal ICCMS ≥1 | QrayCam ΔRmax | 0.665 | 0.663 | -0.003 |
| Proximal ICCMS ≥2 | QrayPen \|ΔFmax\| | 0.847 | 0.843 | -0.004 |
| Proximal ICCMS ≥2 | QrayPen ΔRmax | 0.767 | 0.761 | -0.006 |
| Proximal ICCMS ≥2 | QrayCam \|ΔFmax\| | 0.851 | 0.847 | -0.003 |
| Proximal ICCMS ≥2 | QrayCam ΔRmax | 0.831 | 0.828 | -0.004 |
| Proximal ICCMS ≥3 | QrayPen \|ΔFmax\| | 0.892 | 0.887 | -0.005 |
| Proximal ICCMS ≥3 | QrayPen ΔRmax | 0.767 | 0.756 | -0.012 |
| Proximal ICCMS ≥3 | QrayCam \|ΔFmax\| | 0.908 | 0.905 | -0.003 |
| Proximal ICCMS ≥3 | QrayCam ΔRmax | 0.907 | 0.905 | -0.003 |

*Negative change indicates a lower AUC after exclusion. The largest absolute change was 0.012.*

**Supplementary Table S6. Agreement sensitivity analysis**

| **Analysis** | **Surface** | **n** | **Metric** | **Mean difference (95% cluster CI)** | **95% limits of agreement** | **Within ±5** | **Spearman ρ** |
| --- | --- | --- | --- | --- | --- | --- | --- |
| Primary analysis | Occlusal | 194 | \|ΔFmax\| | 1.15 (-0.28 to 2.63) | -16.30 to 18.61 | 49.5% | 0.901 |
| Primary analysis | Occlusal | 194 | ΔRmax | -2.96 (-5.08 to -0.54) | -32.43 to 26.51 | 50.0% | 0.793 |
| Primary analysis | Proximal | 397 | \|ΔFmax\| | -0.35 (-1.41 to 0.71) | -20.98 to 20.29 | 71.8% | 0.831 |
| Primary analysis | Proximal | 397 | ΔRmax | -3.72 (-14.85 to 2.79) | -197.58 to 190.15 | 81.1% | 0.784 |
| Excluding QrayPen ΔRmax=1962 | Proximal | 396 | \|ΔFmax\| | -0.21 (-1.24 to 0.81) | -20.21 to 19.78 | 72.0% | 0.829 |
| Excluding QrayPen ΔRmax=1962 | Proximal | 396 | ΔRmax | 1.08 (-1.57 to 3.40) | -48.60 to 50.77 | 81.3% | 0.781 |

*Supplementary Figure S1. QLF devices used in the study. (A) QrayCam Pro for quadrant-level imaging. (B) QrayPen for localized tooth-level imaging.*

*Supplementary Figure S2. Representative occlusal cases with white-light, QrayPen, QrayCam, and QA2 ROI displays. The images are illustrative and were not used as separate thresholds.*

*Supplementary Figure S3a. Representative proximal cases with paired QrayPen/QrayCam fluorescence, bitewing radiographs, and QA2 ROI displays. Labels report the original radiographic R-score categories (R1-R2, R4, and R5); the statistical analyses used the collapsed ICCMS-oriented categories defined in Supplementary Table S2.*

*Supplementary Figure S3b. Example QA2 ROI parameter display for one proximal surface.*

*Supplementary Figure S4. Bland-Altman plots for paired QrayCam Pro and QrayPen measurements. Occlusal panels include 194 paired surfaces and proximal panels include 397. All points were retained; the proximal ΔRmax panel includes a central-range inset.*

*Supplementary Figure S5a. Occlusal ROC curves based on 181 surfaces with complete ICDAS scores.*

*Supplementary Figure S5b. Proximal ROC curves based on all 397 surfaces.*
